# An Integrative Approach of an In Vitro Measurement of the Digestibility of Triacylglycerols of Human Milk

**DOI:** 10.3390/molecules26071935

**Published:** 2021-03-30

**Authors:** Antonio Pérez-Gálvez, María Visitación Calvo, Josefa Aguayo-Maldonado, Javier Fontecha

**Affiliations:** 1 Food Phytochemistry Department, Instituto de la Grasa (CSIC), Campus Universitario Building 46, 41013 Sevilla, Spain; 2 Food Lipid Biomarkers and Health Group, Institute of Food Science Research (CIAL, CSIC-UAM), 28049 Madrid, Spain; mv.calvo@csic.es (M.V.C.); j.fontecha@csic.es (J.F.); 3 Neonatology Unit, Hospital Virgen del Rocío, 41013 Sevilla, Spain; pepaaguayo@gmail.com

**Keywords:** human colostrum, milk lipids, in vitro digestion, milk fat globule membrane, bioaccessibility

## Abstract

Several studies have been published regarding the effect of different factors on the digestion of milk lipids, considering their natural structural arrangement as milk fat globules and the efficiency of the digestive enzymes in the lipolysis of such complex structures. During digestion, the lipolytic products are dispersed in vesicles and micelles, which are the source for absorption of digested lipids. Therefore, it is necessary to consider the isolation of the micellar phase from the digesta to appropriately determine the amounts and classes of lipids which are bioaccessible. This study presents an integrative approach that included an isolation procedure to separate the micellar fraction from undigested and non-micellar parts, and the distribution of digested milk lipids in micelles determined directly through chromatographic techniques. Four groups of five full term mothers donated colostrum or mature milk. Two sets of samples were analyzed directly (raw), and two sets were pasteurized and then analyzed. Our data revealed that the profile of digested milk lipids is different depending on the lactation period and processing stage, while the carbon atom number distribution of the digested triacylglycerols in the micellar fraction provides a substantial information regarding the acylglycerols species that are less available for absorption.

## 1. Introduction

The acquisition of the gastrointestinal digestion pattern of human milk lipids is a major challenge because, in addition to the events related to the enzymatic and physicochemical reactions that take place at the lipid–water interface, some other actions must be considered when the target of digestion is the milk fat globule. Hence, the structural arrangement of lipids and proteins in this macrostructure, which is the natural state of milk lipids, remains as a core enriched in triacylglycerols (TAGs), cholesteryl esters and some other apolar lipid categories, surrounded by a trilayer membrane known as the milk fat globule membrane (MFGM). The MFGM contains the characteristic lipid categories, mainly polar lipids and cholesterol, that form cellular membranes, as well as specific membrane proteins [1]. Therefore, the operative events to digest the lipids in such macrostructure should evolve in a different fashion than the digestion of lipid aggregations or homogenous lipid droplets observed in other food categories such as vegetable oils and food emulsions. Indeed, it has been shown that the efficiency of the enzymatic hydrolysis of TAGs of native milk fat vs. homogenized milk fat is different [2]. The reason is that the MFGM might perform a delaying effector in the competence of the hydrolysis of TAGs [3] causing a lag phase in their hydrolysis. To overcome that hindrance, the activity of gastric lipase is crucial by shortening the time of the lag phase and boosting the following intestinal hydrolysis of the TAGs.

Accordingly, a wide number of studies has been published regarding the effect of different factors on structural or compositional changes of the MFGM itself or its immediate environment, which may promote or delay the “dismantling” activity of the polar lipids of the MFGM by pancreatic enzymes, and the lipolytic activity towards the core lipids. Some of those factors are milk homogenization, which produces physical disruption of the MFGM, adsorption of caseins and whey protein on to the surface of milk fat globules [4,5,6]; the effect of bile salts [7] that might remove membrane lipids; the size of milk fat globules and the flocculation phenomena observed during the early stages of digestion [7,8,9]; and the intricate effects of technological processes, including pasteurization and high pressure homogenization, on the quality of the interface [8,10,11]. These studies revealed that although the MFGM could a priori be envisioned as a barrier to the lipolytic activity of pancreatic secretions, indeed it is a structure that enhances the interfacial events involving lipases and bile salts. Any process remodeling or suppressing the MFGM produces a negative effect on the efficiency of the hydrolysis of TAGs contained in the core of the milk fat globules.

Consequently, this direct influence of the MFGM in the nutritional efficiency of milk lipids is one of the reasons that promotes the study of the qualitative and quantitative composition of lipids in the MFGM, and how they are organized and edge the membrane proteins. In fact, the demonstration of the lateral heterogeneity in the ordering of polar lipids and cholesterol in the MFGM, by using confocal laser scanning microscopy and labelling of the membrane with a fluorescent probe, specifically reveals the presence of dispersed sphingolipids liquid ordered domains featured by different sizes and shapes, providing the MFGM with stability and rigidity [12]. Hence, it has been suggested that such rigid microdomains develop a functional role in the digestibility of the MFGM and milk lipids [12,13]. Further, they could function as sites for the interactions with the brush border membrane vesicles (BBMV) containing enzymes secreted by the intestinal epithelium like brush border alkaline sphingomyelinase and neutral ceramidase, which are specifically present in BBMV and responsible for the hydrolysis of sphingolipids [14].

In addition to the non-nutritious functionality of the MFGM as a biophysical structure, we also claim its role as a source of polar lipids, including phosphatidylcholine (PC) phosphatidylethanolamine (PE), phosphatidylserine (PS) and phosphatidylinositol (PI) as well as sphingolipids, sphingomyelin (SM), and glycosphingolipids, cerebrosides and gangliosides, being the latter quantitatively minor compounds in the MFGM [15,16,17]. These polar lipids show significant actions in addition to their role as essential constituents of membranes, such as transmembrane signaling, lipid-cholesterol transport and metabolism, and brain development [14,18], while PC and SM are significant sources of choline for the newborn [19,20]. Therefore, determination of bioavailability of lipids from milk fat globule should not only encompass efficiency of lipolysis of TAGs but also how the lipids from the MFGM become bioaccessible. However, the methods applied so far to measure the digestibility of milk lipids were designed to probe the mode of action of pancreatic lipase and its interactions with colipase and bile salts [21], and the impact in the TAGs hydrolysis of the particular organization of lipids in the MFGM, that is, size and interfacial area, surface composition and organization, as well as the structural features of the TAGs [9]. Thus, the common in vitro experimental approach consists of the incubation of the milk sample with gastric and/or pancreatic secretions, i.e., gastric lipase, pepsin, lipase and bile salts. The amount of free fatty acids released from the enzymatic activity is measured using NaOH in the 0.05–0.1 M range through pH-stat titrimetry. This measurement is accompanied in some studies with GC analysis of the lipids (free fatty acids, monoacylglycerols, diacylglycerols and TAGs) isolated through thin layer chromatography from aliquots separated from the digestive tests [9,22].

These methods have successfully revealed the biochemical steps of milk lipids hydrolysis but much less attention has been paid to the physical-chemical events involved in lipid digestion. During digestion, the lipolytic products are primarily dispersed in vesicles and micelles, and the latter are the source from which absorption of digested lipids preferentially takes place [23,24]. Two factors were selected to ascertain whether our protocol can measure effects in lipid species incorporated in mixed micelles after digestion. The nature of the milk secretion (colostrum vs. mature milk) and application of pasteurization to the milk secretion (raw vs. pasteurized milk), as it has been demonstrated that both factors influence the result of lipid digestion [8,10,11,25]. Therefore, it would be required to consider the isolation of the micellar phase from the digesta to appropriately determine qualitatively and quantitatively the bioaccessible lipids. Accordingly, the aim of this study was to qualitatively characterize the micellar lipids after the application of an in vitro digestion protocol to human milk. Therefore, we present an integrative approach that comprised an isolation procedure of the micellar fraction and non-micellar fraction from undigested parts, and the distribution of digested lipids in micelles determined directly through chromatographic techniques.

## 2. Results

**Figure 1:**
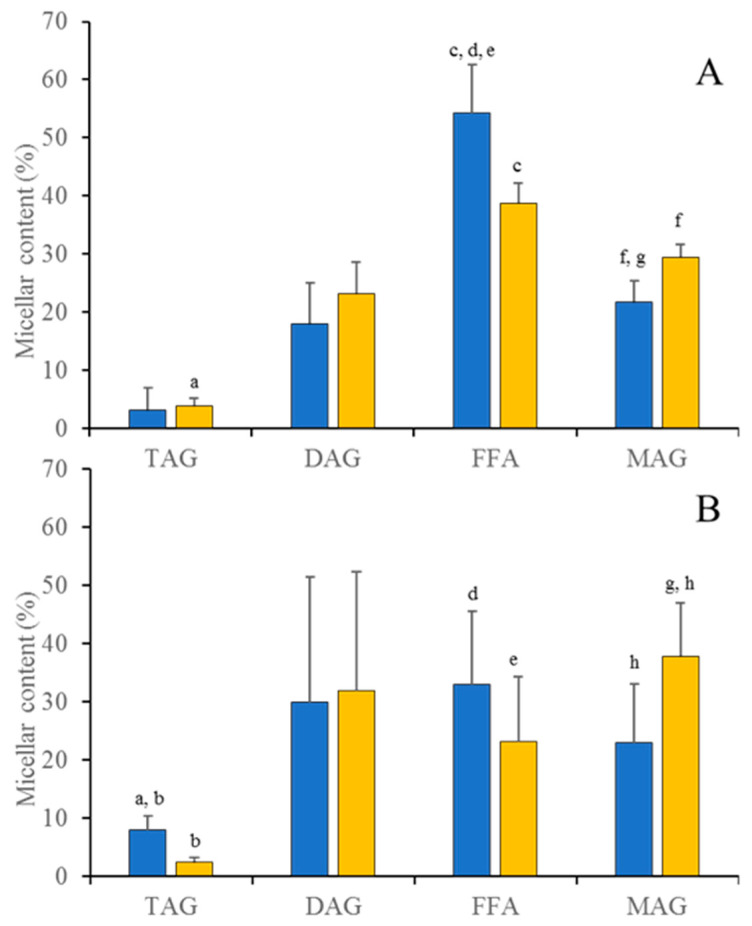
Distribution of the milk acylglycerols in micelles after the application of the in vitro digestion protocol in (**A**) human colostrum and in (**B**) mature milk, (raw sample ■ and pasteurized sample ■). Bars represent median values and whiskers stand for standard deviation. Significant differences were observed for each lipid class, independently of the lactation stage and processing stage, and denoted with the same superscript letter.

In this study, we have not only considered how the lipolytic products were released from the milk fat globules, but also their incorporation into micelles, which are the aggregated structures with different size and composition that perform a reservoir-like action to replete the apical plasma membrane of the epithelial cells with milk lipid molecules that are subsequently absorbed. Table 1 shows the content in TAGs, diacylglycerols (DAGs), monoacylglycerols (MAGs) and free fatty acids (FFAs) of the human milk samples, while the profile of these lipid species in the micelles once the samples were digested is represented in Figure 1.

As expected, TAGs were the main lipid category in the predigested samples, while FFAs, DAGs and MAGs were present in the micellar fraction within variable percentages (20–50%). Undigested TAGs were still observed in the micelles although at lower percentages (2.5–8%). This behavior was denoted both for raw samples of human colostrum (HC) and mature milk (HM) samples. However, there was a heterogeneous profile of the digested lipid forms in raw HC (Figure 1A) with a higher trend to accumulate in the micellar fraction FFAs (54.2%), which was significantly different (*p* = 0.03) than the following lipid classes, MAGs (21.8%) and DAGs (18.1%), data that were not significantly different (*p* = 0.35), while the digested raw HM samples accumulated those lipid species in a more homogenous distribution (32.9%, 22.9% and 29.9% for FFAs, MAGs and DAGs, respectively, Figure 1B), data that were not significantly different (*p* = 0.69). This heterogenous to homogenous shift from HC to HM samples was also observed for the pasteurized samples (Figure 1A,B). The digested pasteurized HC samples distributed the lipid categories in the micelles with a higher contribution for the FFAs (38.7%), then the MAGs (29.5%) and finally the DAGs (23.2%), data that were not significantly different (*p* = 0.14), while the digested pasteurized HM samples reached very similar percentage values for these lipid classes (23.1%, 37.9% and 31.9% for FFAs, MAGs and DAGs, respectively, Figure 1B, *p* = 0.69).

Here we noticed an interesting result that is related to both the effect of the pasteurization on the progress of the digestion and the method applied to measure such progress. First, there was a trend for a lower accumulation of FFAs in the micellar fraction when the digestion was performed with the pasteurized samples both for the HC and the HM (although that effect was only significant for HC, Figure 1A). We also compared the efficiency of the micellarization process of each lipid class (TAGs, DAGs, FFAs or MAGs) among all types of sample. Table 2 contains the *p* values for the Mann–Whitney test, so that it could be noted those lipid classes where their micellar content was significantly different or not. Differences are marked in Figure 1. Micellar FFAs reached the highest value on the raw HC, while the micellar FFAs contents for the raw and pasteurized HM and pasteurized HC were within the same range values and not significantly different. This result correlated with the micellar contents of MAGs, where we denoted the highest values for the pasteurized samples (HC and HM), while the DAGs fractions were equally micellarized, independently of the lactation period or the processing stage of the samples.

**Figure 2:**
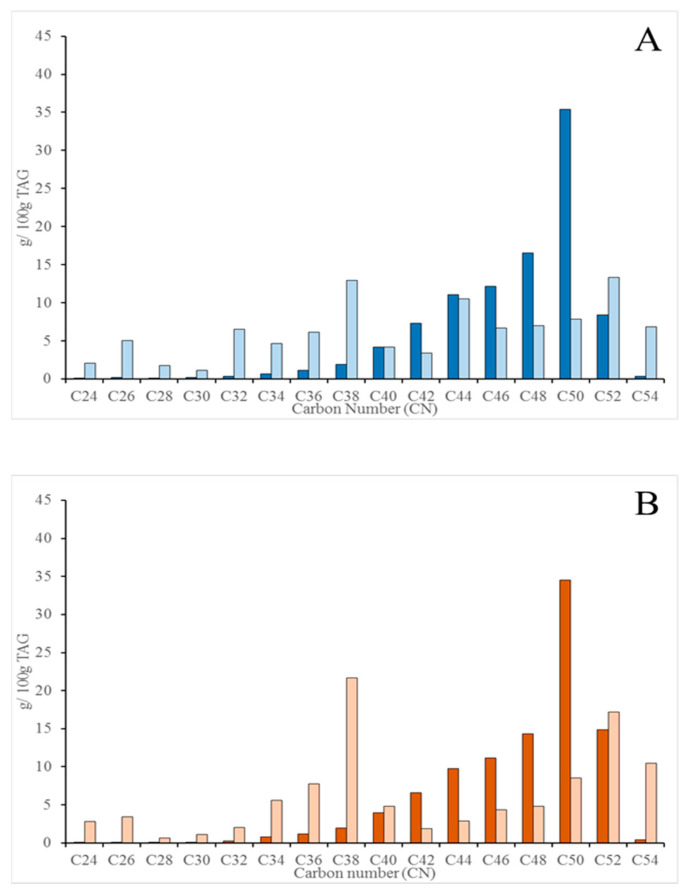
Distribution of the acylglycerols molecular species (%), in HC samples, according to their carbon atom number (CN). (**A**) raw HC samples (undigested ■ and digested ■). (**B**) pasteurized HC samples (undigested ■ and digested ■). Significant differences were observed when each pair of CN value was compared except for the CN40 and CN52.

**Figure 3:**
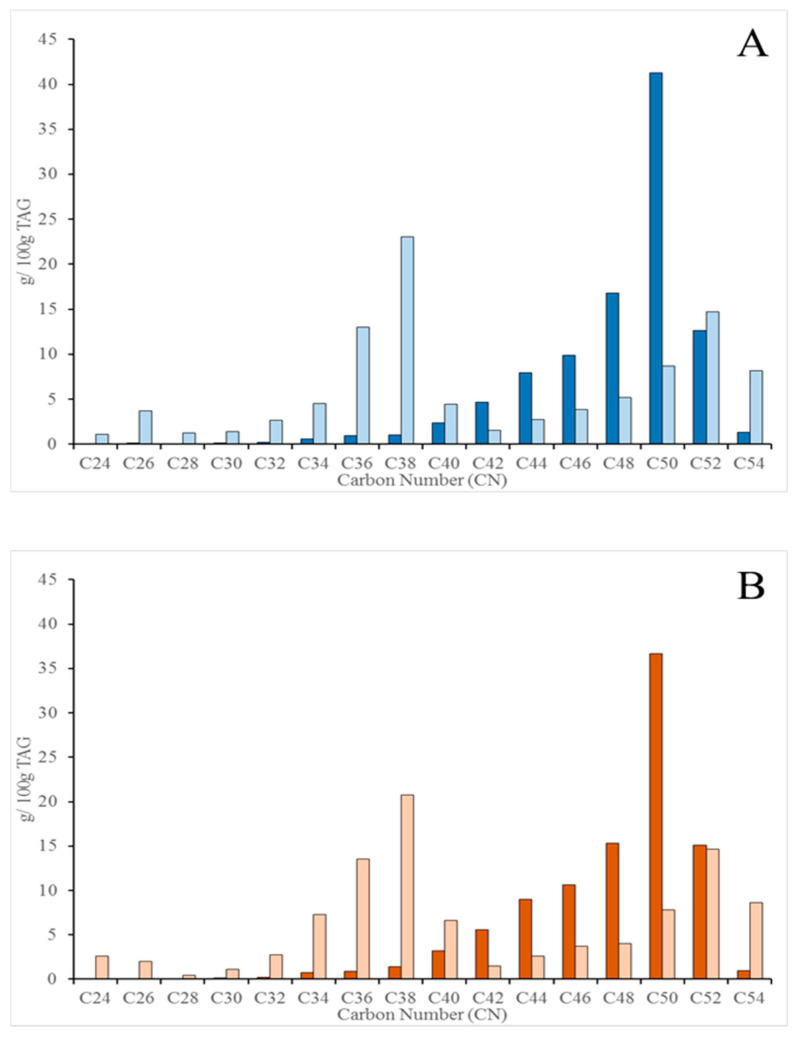
Distribution of the acylglycerols molecular species (%) in HM samples, according to their CN. (**A**) raw HM samples (undigested ■ and digested ■). (**B**) pasteurized HM samples (undigested ■ and digested ■). Significant differences were observed when each pair of CN value was compared except for the CN40 and CN52.

Distribution of the acylglycerol molecular species according to their carbon atom number (CN) was also analyzed and is shown in Figure 2A and Figure 3A for the raw HC and HM samples, respectively, before and after the application of the in vitro digestion protocol. The undigested samples for HC and HM showed the typical monomodal trend in the CN of the acylglycerols [26], with a significant higher contribution of the long chain species (CN48-52) to the profile. However, the CN pattern for the raw HC and HM samples, once they were digested, showed a higher contribution of the CN34-40 species. Although the method applied does not allow to distinguish TAGs from DAGs, the increments in the CN34-40 of the digested samples should mainly be attributed to the generation of DAGs, while some of the TAGs with long chain fatty acids were still contributing to the CN profile of the micelles. This was particularly true for the digested HM samples (Figure 3A) where the trend towards CN34-40 was not prevalent. The monomodal trend for the CN distribution in the raw samples and the shift to lower CN values in the micellar fraction was independent of the lactation period (colostrum or mature milk). This information is relevant as that is the bioaccessible pattern that should be reproduced by the digested infant formulae. Pasteurized samples followed the same pattern (Figure 2B and Figure 3B) both for the raw and digested TAGs. Even, the weak trend denoted above for the digested raw HM samples towards the CN34-40 (Figure 3A) was overcame in the digested pasteurized counterparts. The hydrolysis of the TAGs took place in a similar fashion as it was observed for the raw samples, yielding the same CN distribution pattern. The CN40 and CN52 values were the exceptions among milk acylglycerols that was equally distributed in undigested milk (both raw and pasteurized HC) and micelles, that is, the digestion process did not change the CN40 and CN52 contribution to the CN profile.

## 3. Discussion

The available evidence regarding the impact of the MFGM in the fate of core lipids during digestion is providing a significant knowledge about the mechanisms and efficient activity of enzymes present in the digestive secretions, and the cooperative action with other gastric and luminal components. While this knowledge may contribute to design infant milk formula resembling the original structures of human milk [27], by showing that this concept is feasible and nutritionally efficient, key pieces of information are still necessary. One of these pieces is how the dynamics of the digestion during the progress of lactation should perform and influence the micellarization. In a previous work we already observe this different behavior regarding the efficiency of the accumulation of lipid categories in the micellar fraction depending on the lactation stage [28]. Hence, micellarization of carotenoids is related to the lactation period, with a heterogenous profile of micellar carotenoids resulting from the in vitro digestion of HC samples. Considering the results presented in this study, that influence is extended to other lipid categories of the milk fat globules, as the lipolytic pattern is affected by the lactation stage (colostrum vs. mature milk).

Regarding the influence of the processing stage, our results correlate well with other studies where the effect of pasteurization on the lipolysis of milk fat is determined [9,10]. These studies recognize the quantity and quality of the interface as important parameters for the lipolytic kinetics of the milk fat globule, producing a lack of improvement of digestibility of milk samples subjected to thermal processing. Besides, when only the FFAs fraction is measured, another piece of information is missing as it was denoted here, that other lipid categories yielded from digestion were also accumulated in the micelles and they became bioaccessible. Although the micellarization of FFAs species was negatively affected by the pasteurization (Figure 1A,B), it was also true that there was a trend for a higher accumulation of MAGs in the micelles of the pasteurized samples in comparison with the values observed for the raw samples (both for HC and HM). Thus, the micellar content of the MAGs in pasteurized HC was significantly higher than the corresponding value of raw HC, and the same effect was denoted for the micellar MAGs on the raw and pasteurized HM samples.

Another point that should be remarked is that the analysis of the acylglycerols molecular species by CN distribution in the micellar fraction reveals whether the enzymatic lipolysis was effective or not toward any of the acylglycerols species, and our data revealed one exception that should be considered in future studies. The CN52 was particularly recalcitrant to the enzymatic hydrolysis as the distribution in the micellar fraction was the same as in the undigested samples, a result independent of the lactation period and processing stage. CN52 species could represent nutritionally interesting combinations of different fatty acids in the acylglycerols. Particularly, a recent study where the fatty acid regioisomer distribution in human milk TAGs was characterized [29] has shown that C18:C16:C18 is the prevalent distribution for the CN52, that is, palmitic acid in the *sn*-2 position. The lack of hydrolysis of the CN52 observed in our study could lead to thinking that the availability of monopalmitoylglycerol is compromised. However, the CN52 is not the single source of monopalmitoylglycerol, as 70–80% of the palmitic acid is in the *sn*-2 position [30,31] and other species, CN50 and CN48 that in the samples analyzed our study where most abundant than CN52, should guarantee the availability of monopalmitoyglycerol.

The characterization of milk lipid species in mixed micelles beyond the FFA content is useful to improve the knowledge of those TAGs species that yield available digestive products (MAGs and DAGs) for assimilation. Thus, infant-fed formula with TAGs containing monounsaturated or polyunsaturated fatty acids distributed preferentially in the *sn*-2 position are of interest as the corresponding MAGs are potentially ready for absorption. Our data regarding the CN distribution, and the presence of DAGs species accumulated in the micellar fraction after digestion, would also expand the range of other TAGs with specific fatty acids, not only at *sn*-2 but also at *sn*-1 or *sn*-3 that are also more available, although it is required to determine their precise fatty acid composition. Nevertheless, there is still no agreement on the recommendations regarding any long chain fatty acids content and specifications for the *sn* position of these fatty acids in TAGs [32].

## 4. Materials and Methods

### 4.1. Subjects

The study population comprised 20 full term mothers (gestational age > 37 weeks) who donated colostrum (3–5 days after delivery) or mature milk (30 days after delivery). Eligible participants were nonsmoking mothers with no chronic diseases. Exclusion criteria applied were pathologies or infections during the gestation, developmental anomalies in the fetus or death of the child. Mothers following any special diets, such as vegetarians or those taking any prenatal supplements, were not included. Participants provided informed consent for inclusion before they participated in the study, which was conducted in accordance with the Declaration of Helsinki (https://www.wma.net/policy/current-policies/ accessed on 29 March 2012). The study protocol was approved by the Ethics Committee of the Hospital Universitario Virgen del Rocío (AGL2017-87884-R).

### 4.2. Samples

The human milk samples were collected at the Neonatology Unity of the Hospital Universitario Virgen del Rocío (Sevilla, Spain). Milk samples were obtained by collection of the total milk volume of one breast during one milk expression session into a polypropylene bottle. Milk collection was recommended in the morning 2 to 4 h after the previous breastfeeding. We established 2 groups (5 samples per group) of mothers donating colostrum or mature milk, and 2 groups (5 samples per group) of mothers donating colostrum or mature milk which were subjected to Holder pasteurization (62.5 °C, 30 min). Raw and pasteurized samples were stored at 4 °C and transported to the laboratory. Subsequent analyses of the samples, that is, application of the in vitro digestion protocol, were performed within 1 day.

### 4.3. Reagents and Solvents

Pepsin from porcine gastric mucosa, porcine bile extract and pancreatin and lipase from porcine pancreas were obtained from Sigma (St. Louis, MO, USA). Gastric lipase (rabbit gastric extract, RGE 15) was obtained from Lipolytech^®^ (Marseille, France). The protocols described in Brodkorb et al. [28] were applied to determine the enzyme activities. Dichloromethane, chloroform, hexane, methanol, isooctane, isopropanol, dimethylformamide and acetonitrile, which were all HPLC grade, were purchased from Labscan (Dublin, Ireland). The purified water was obtained from a Milli-Q water purification system (Millipore, Milford, MA, USA). Sodium sulphate anhydrous, sodium chloride, potassium chloride and sodium carbonate were obtained from Panreac (Barcelona, Spain). Sodium methoxide (95%), sodium citrate dehydrate, formic acid (98%), triethylamine (99.5%), acetic acid and trinanoin were supplied by Sigma-Aldrich (St. Louis, MO, USA).

### 4.4. In Vitro Digestion of Human Milk

The experimental conditions described by Ménard et al. [22] were applied with slight modifications already published [24,25]. Briefly, the in vitro protocol does not include the oral phase, while the gastric and intestinal steps mimic the digestive conditions of full-term infants; gastric phase: Higher ratio (*v*/*v*) of milk to gastric secretions (63:37 vs. 50:50 established for adults), with lower pepsin enzyme activity per mL of gastric fluid (268 U/mL vs. 2000 U/mL) and higher gastric pH value (5.3 vs. 3), while the intestinal phase follows a higher ratio (*v/v*) of milk to gastric plus intestinal secretions (39:61 vs. 25:75 established for adults), with lower lipase and bile salt contents at the intestinal fluid (90 U/mL and 3.1 mmol/L vs. 2000 U/mL and 10 mmol/L established for adults) and a slightly lower pH (6.6 vs. 7 established for adults). Mature milk sample (6 mL) was mixed with 3.5 mL of gastric fluid and incubated for 60 min under shaking (140 rpm) in a thermostatically controlled cabinet (TC 135 S, Tintometer GmbH, Dortmund, Germany) at 37 °C. The composition of the gastric fluid was 94 mM NaCl, 13 mM KCl at pH 5.3 (HCl 1 M). The pool of gastric fluid enzymes included pepsin (268 U/mL) and gastric lipase (19 U/mL). Once the gastric phase ended, the sample was cooled in water, pH adjusted to 6.6 and mixed with 5.5 mL of intestinal fluid. The intestinal fluid was composed of 85 mM NaHCO_3_, 164 mM NaCl, and 10 mM KCl, while the bile extract provided with 3.1 mM bile salt concentration at pH 7. Porcine pancreatin, with 90 U/mL of lipase activity, was added to the intestinal fluid. The resulting cocktail was incubated at 37 °C with shaking for 60 min. Once the in vitro digestion is completed, the total digesta contains a suspension of remaining undigested milk lipids and proteins in the aqueous phase that also includes the mixed micelles where the digested lipids accessible for assimilation are arranged. To separate the mixed micelles, we applied a low-speed centrifugation procedure that yields a clarified aqueous phase separated from oil droplets. Thus, the complete digesta volume (15 mL) was centrifuged (12,000× *g*, 5 min, 4 °C) in an Avanti^TM^ J-25 centrifuge (Beckman Coulter^TM^, Brea, CA, USA) equipped with a Beckman model JA-25.50 rotor (Kildare, Ireland). The micellar fraction (5 mL) was collected with a syringe attached to a needle (0.6 by 80 mm, 23 G × 3⅛”) carefully avoiding the disruption of the upper oily phase (1 mL) and the pelleted undigested material [24,33]. Micellar fraction was used for measurement of the micellar lipid categories. Three replicates of the in vitro digestion procedure for each sample were carried out.

### 4.5. Analysis of Lipids

The methodologies applied for the extraction of the fat content and analysis of the lipid categories have recently been published by Calvo et al. [34]. Briefly, the lipids were isolated from the milk samples or from the micellar fraction by the Löfgren et al. [35] method with some modifications based on the optimization of solvent/ratio sample. The determination of TAGs by GC-FID was performed on a Clarus 400 GC (PerkinElmer Ltd., Beaconsfield, UK) equipped with an automatic split/splitless injector and a flame ionization detector. The experimental chromatographic conditions were the same as published by Fontecha et al. [36]. For qualitative and quantitative analysis of TAG, response factors were calculated using an anhydrous milk fat (reference material BCR-519; EU Commission, Brussels, Belgium; purchased from Fedelco Inc., Madrid, Spain) of known TAG content, and glyceryl trinanoate as internal standard (100 μL; 1 mg/mL). Separation of lipid classes was accomplished by using an HPLC system (model 1260; Agilent Technologies Inc. Palo Alto, CA, USA) coupled with an ELSD (SEDEX 85 model; Sedere SAS, Alfortville, Cedex, France) as described by Castro–Gómez et al. [37]. Assays were carried out in triplicate.

### 4.6. Statistical Analysis

Data are reported as the median values, with the 25th and 75th percentiles. Considering the non-normality of data (Shapiro–Wilk test) and the lack of homoscedasticity (Levene test), we applied a non-parametric statistical procedure in the SPSS software (version 25 IBM^®^ Corp., SPSS^®^ Statistics version 26, IBM, New York, NY, USA). Comparisons between data of lipid families within the same type of sample were performed with the Wilcoxon test. The Kruskal–Wallis test was applied to analyze the differences in the contents of lipids categories of the milk samples, considering the lactation period (colostrum or mature milk) and the processing stage (raw or pasteurized), while the Mann–Whitney test was applied to denote those pairs of lipid categories with significant differences. The significance was set at *p* < 0.05.

## 5. Conclusions

The application of an in vitro digestion protocol tailored for simulating infant digestion and the isolation of the micellar fraction to analyze the digestive products of TAGs is the suitable strategy to predict the influence of endogenous (MFGM composition) and exogenous (processing techniques) factors in the quality of the lipid profile that becomes bioaccessible to the intestinal epithelial cells. This work presents a procedure to describe those lipid species that are accumulated in mixed micelles and the effect(s) of endogenous and exogenous factors in the efficiency of the intestinal hydrolysis of TAGs and the subsequent micellar accumulation. Thus, new knowledge of a complex process such as human digestion has been described, and also that not only the exact determination of the individual TAGs species is required, but also that the isolation of the micellar contents is the approach to determine which ones are prone to become accessible.

## Figures and Tables

**Table 1:** Distribution of lipid categories in human colostrum and mature milk samples used in the study. Data are expressed as percentage (median values, n = 5, including 25th and 75th percentiles), for human colostrum (HC) or mature milk (HM) both raw and pasteurized.

Lipid Class	Raw HC	Raw HM	Pasteurized HC	Pasteurized HM
TAGs	97.2 (94.0; 97.8)	96.2 (96.0; 96.7) ^1^	99.4 (98.1; 99.5) ^1^	99.1 (98.3; 99.2)
DAGs	2.27 (1.72; 4.35) ^1^	2.77 (2.38; 2.89) ^2^	0.38 (0.37; 1.19) ^1,2^	0.67 (0.66; 1.51)
MAGs	0.03 (0.02; 0.28)	0.09 (0.07; 0.20)	-	-
FFAs	0.34 (0.27; 1.06) ^1^	0.48 (0.32; 1.11) ^2^	0.10 (0.09; 0.14)	0.09 (0.09; 0.12) ^1,2^

Data with significant differences are denoted with the same superscript number. Comparisons are made within each lipid class (Kruskal–Wallis test, *p* < 0.05).

**Table 2:** *p* values for the comparison (Mann–Whitney U test) between raw and pasteurized HC and HM samples. Comparisons are made within each lipid category (triacylglycerols (TAGs), diacylglycerols (DAGs), free fatty acids (FFAs) and monoacylglycerols (MAGs)) value observed in the micellar content.

	TAGs				DAGs				FFAs				MAGs			
	Raw HC	Past. HC	Raw HM	Past. HM	Raw HC	Past. HC	Raw HM	Past. HM	Raw HC	Past. HC	Raw HM	Past. HM	Raw HC	Past. HC	Raw HM	Past. HM
Raw HC	-	0.15	0.10	0.42	-	0.22	0.55	0.42	-	0.03	0.02	0.01	-	0.02	0.70	0.03
Past. HC	0.15	-	0.03	0.06	0.22	-	1.00	0.84	0.03	-	0.22	0.06	0.02	-	0.42	0.15
Raw HM	0.10	0.03	-	0.01	0.55	1.00	-	0.84	0.02	0.22	-	0.31	0.70	0.42	-	0.04
Past. HM	0.42	0.06	0.01	-	0.42	0.84	0.84	-	0.01	0.06	0.31	-	0.03	0.15	0.04	-

## Data Availability

The data presented in this study are available on request from the corresponding author. The data are not publicly available due to privacy.

## Sample Availability

Not available.
